# Two Novel Sets of Genes Essential for Nicotine Degradation by *Sphingomonas melonis* TY

**DOI:** 10.3389/fmicb.2016.02060

**Published:** 2017-01-17

**Authors:** Haixia Wang, Cuixiao Xie, Panpan Zhu, Ning-Yi Zhou, Zhenmei Lu

**Affiliations:** ^1^Institute of Microbiology, College of Life Sciences, Zhejiang UniversityHangzhou, China; ^2^State Key Laboratory of Microbial Metabolism, School of Life Sciences and Biotechnology, Shanghai Jiao Tong UniversityShanghai, China

**Keywords:** nicotine, *Sphingomonas melonis* TY, *ndrA1A2A3*, *ndrB1B2B3B4* and nicotine dehydrogenase

## Abstract

Nicotine is a type of environmental pollutant present in the tobacco waste that is generated during tobacco manufacturing. *Sphingomonas melonis* TY can utilize nicotine as a sole source of carbon, nitrogen and energy via a variant of the pyridine and pyrrolidine pathway (the VPP pathway). In this study, we report the identification of two novel sets of genes, *ndrA1A2A3*, and *ndrB1B2B3B4*, which are crucial for nicotine degradation by strain TY. *ndrA1A2A3* and *ndrB1B2B3B4* exhibit similarity with both nicotine dehydrogenase *ndh* from *Arthrobacter nicotinovorans* and nicotine hydroxylase *vppA* from *Ochrobactrum* sp. SJY1. The transcriptional levels of *ndrA1A2A3* and *ndrB1B2B3B4* in strain TY were significantly upregulated in the presence of nicotine. Furthermore, *ndrA1* or *ndrB2* knockout resulted in a loss of the ability to degrade nicotine, whereas gene complementation restored the capacity of each mutant to utilize nicotine for growth. Biodegradation assays indicated that the mutant strains retained the ability to degrade the first intermediate in the pathway, 6-hydroxynicotine (6 HN). However, heterologous expression of *ndrA1A2A3* and *ndrB1B2B3B4* did not confer nicotine dehydrogenase activity to *E. coli* DH5α, *Pseudomonas putida* KT2440 or *Sphingomonas aquatilis*. These results provide information on the VPP pathway of nicotine degradation in *S. melonis* TY, and we conclude that these two sets of genes have essential functions in the conversion of nicotine to 6 HN in strain TY.

## Introduction

Tobacco products are among the most widely popular goods worldwide due to the addictive component in tobacco: nicotine. Nicotine is the most abundant alkaloid in tobacco plants, and a large amount of tobacco waste produced every year is disseminated in the environment through water and soil. Nicotine is very harmful to public health because it can be easily absorbed and can pass the blood-brain barrier (O'neill et al., 2002; Lemay et al., 2004).

As important decomposers in ecosystems, microbes possess powerful, and versatile degradation capacities, as reflected in the type of substrates utilized, the metabolic mechanisms involved, and diversity of catalyzing enzymes. One example is the diversity in metabolic pathways and molecular mechanisms of nicotine degradation by microorganisms. Research on the microbial degradation of nicotine has been studied since the 1950s (Wada and Yamasaki, 1953). To date, various bacteria and fungi are reported to be able to decompose nicotine through at least six pathways, though there are four main degradation pathways. (1) The pyridine pathway, which begins with the hydroxylation of the nicotine pyridine ring to generate 6-hydroxynicotine (6 HN), and then 6-hydroxy-N-methylmyosmine, 6-hydroxypseudooxynicotine (6 HPON), and 2, 6-dihydroxy-pseudooxynicotine, and generates nicotine blue and succinic acid at last, has been described in *Arthrobacter* (Hochstein and Rittenberg, 1959a,b, 1960; Richardson and Rittenberg, 1961a,b; Gherna et al., 1965; Holmes and Rittenberg, 1972; Holmes et al., 1972; Chiribau et al., 2006), *Nocardioides* (Ganas et al., 2008) and *Rhodococcus* (Cobzaru et al., 2011). And the most studied metabolic pathway of the pyridine pathway was encoded by megaplasmid pAO1 of *Arthrobacter nicotinovorans* (Brandsch, 2006), for which every enzyme involved has been validated. (2) The pyrrolidine pathway, representative of *Pseudomonas*, begins with oxidation of the pyrrolidine ring to produce N-methylmyosmine, which leads to formation of succinate and 2, 5-dihydroxypyridine (2, 5-DHP); the latter then enters the maleamic acid pathway (Wang et al., 2007; Tang et al., 2012). This pathway has been comprehensively studied in *Pseudomonas putida* S16, and the final steps are catalyzed sequentially by DHP dioxygenase (Hpo), NFM deformylase (Nfo), maleamate amidase (Ami) and maleate isomerase (Iso) to produce fumaric acid (Tang et al., 2012). (3) The methyl pathway, which is found in some fungi such as *Aspergillus* and *Pellicularia*, degrades nicotine via demethylation to nornicotine (Uchida et al., 1983; Meng et al., 2010), while the pathway has not been well-elucidated. (4) A variant of the pyridine and pyrrolidine (VPP) pathway constitutes the fourth main pathway of nicotine degradation. Occurring in several strains, the newly identified VPP pathway first transforms nicotine into 6 HPON through 6-hydroxy-L-nicotine and 6-hydroxy-N-methylmyosmine via the pyridine pathway; the intermediates then enter the pyrrolidine pathway followed by formation of 6-hydroxy-3-succinoylpyridine and 2, 5-DHP. This VPP pathway has been described in *Agrobacterium tumefaciens* strain S33, *Shinella* sp. HZN7, and *Ochrobactrum* sp. strain SJY1 (Wang et al., 2012; Ma et al., 2013; Yu et al., 2014). Surprisingly, a newly isolated strain, *Pusillimonas* sp. T2, was found to possess both the VPP pathway and a partial pyridine pathway with 2, 6-dihydroxypyridine formation (Ma et al., 2014). Many intermediates, such as 6-hydroxy-3-succinoylpyridine, generated through these diverse nicotine metabolic pathways can potentially be transformed into commercially valuable compounds such as analogs of epibatidine (Wang et al., 2005). In addition, the various nicotine metabolic pathways indicate the diversity of the mechanisms responsible. Although *Sphingomonas melonis* strain TY, isolated by our research team, can degrade nicotine efficiently, the genes involved are unclear and may differ from the genes described thus far (Wang et al., 2011).

In this work, we studied the nicotine-degrading *S. melonis* strain TY, a new bacterium able to degrade nicotine via the VPP pathway. The draft genome sequence of *S. melonis* TY was obtained using the Illumina approach. Sequence analysis revealed two sets of putative nicotine metabolic genes that were found to be essential for nicotine dehydrogenase in strain TY.

## Materials and methods

### Chemicals and reagents

(*S*)–(-)-Nicotine (>99%) was obtained from Chemsky international Co., Ltd (Shanghai, China). 6 HN was a gift from Jiguo Qiu (Nanjing Agricultural University, China). TransStart® FastPfu DNA Polymerase for fragment amplification was purchased from TransGen Biotech (Beijing, China). Restriction enzymes used for plasmid construction and a premixed protein marker for protein electrophoresis were purchased from Takara Biotechnology Co., Ltd. (Dalian, China). Antibiotics, isopropyl β-D-1-thiogalactopyranoside (IPTG) and other reagents were purchased from Shanghai Sangon Biotech Co., Ltd. (Shanghai, China). A plasmid extraction kit, gel extraction kit and DNA purification kit were obtained from Omega Bio-tek, Inc (Norcross, GA, USA).

### PCR programs used in this study

PCR program used for high-fidelity DNA polymerase was according to the manual: pre-denaturing at 95°C for 2 min; denaturing at 95°C for 20 s, annealing at certain temperature (according to the primer pair) for 20 s, extending at 72°C at a speed of 2 kb/min, 32 cycles; extending at 72°C for 5 min; keeping at 4°C. General PCR program used for fragment amplification was pre-denaturing at 94°C for 5 min; denaturing at 94°C for 30 s, annealing at certain temperature (according to the primer pair) for 30 s, extending at 72°C at a speed of 1 kb/min, 32 cycles; extending at 72°C for 5 min, keeping at 4°C. PCR program used for reverse transcription quantitative PCR (RT-qPCR) was: pre-denaturing at 94°C for 30 s; denaturing at 94°C for 5 s, annealing at 60°C for 15 s, extending at 72°C for 12 s, 40 cycles.

### Bacterial strains, plasmids, and growth conditions

The bacterial strains and plasmids used in this study are listed in Table 1; the primers used are provided in Table S1. Wild-type *S. melonis* TY (collection number CGMCC 1.15791) can utilize nicotine as a sole source of carbon, nitrogen and energy for growth (Wang et al., 2011). Wild-type TY and derivatives were cultured aerobically at 30°C in LB medium or inorganic salt medium (ISM) supplemented with nicotine, as described previously (Yang, 2010). *Escherichia coli* strains were grown in LB broth at 37°C. When necessary, kanamycin and tetracycline were used at final concentrations of 50 and 10 μg/mL, respectively. IPTG was used to induce expression at gradient concentration, and 2, 6-diaminopimelic acid (2, 6-DAP) was used at a final concentration of 0.3 mM for *E. coli* WM3064.

**Table 1:** **Strains and plasmids used in this study**.

**Strain or plasmid**	**Relevant characteristics**	**References or source**
**STRAINS**		
*Escherichia coli*		
DH5α	*supE44 lacU169*(Φ80dlacZΔM15)*hsdR17 recA1 endA1 gyrA96Δthi relA1*	Woodcock et al., 1989
DH5α pRK415 *ndrB1B2B3B4orf1orf2*	DH5α transformed with pRK415*-ndrB1B2B3B4orf1orf2*, Tc^R^	This study
DH5αpRK415	DH5α transformed with pRK415, Tc^R^	This study
WM3064	Donor strain for conjugation, 2,6-diaminopimelic acid auxotroph: *thrB1*004 *pro thi rpsL hsdS lacZ*ΔM15 RP4-1360 Δ(*araBAD*)*567* Δ*dapA*1341::[*erm pir*(wt)]	Dehio and Meyer, 1997; Saltikov and Newman, 2003
BL21(DE3)	F^−^*ompT hsdS_B_* (${\text{r}}_{\text{B}}^{-}$ ${\text{m}}_{\text{B}}^{-}$)*gal dcm lacY1*(DE3)	Transgen
BL21(DE3)pET28a-*ndrB1B2B3B4orf1orf2*	BL21(DE3) transformed with pET28a-*ndrB1B2B3B4orf1orf2*, Kan^R^	This study
BL21(DE3)pET28a	BL21(DE3) transformed with pET-28a(+), Kan^R^	This study
***Sphingomonas*** **SPECIES**		
TY	Wild type, nicotine-degrading strain, G^−^, Amp^R^, Kan^S^, Tc^S^	This study
TYΔ*ndrA1*	*Sphingomonas melonis* TY mutant with *ndrA1* gene replaced by kanamycin resistance gene from plasposon pTnMod-Okm, Amp^R^, Kan^R^	This study
TYΔ*ndrB2*	*Sphingomonas melonis* TY mutant with *ndrB2* gene replaced by kanamycin resistance gene from plasposon pTnMod-Okm, Amp^R^, Kan^R^	This study
TYΔ*ndrA1*(pRK415-*ndrA1*)	*ndrA1* gene was complemented by pRK415-*ndrA1* in *Sphingomonas melonis* TYΔ*ndrA1*, Amp^R^, Kan^R^, Tc^R^	This study
TYΔ*ndrB2*(pRK415-*ndrB2*)	*ndrB2* gene was complemented by pRK415-*ndrB2* in *Sphingomonas melonis* TYΔ*ndrB2*, Amp^R^, Kan^R^, Tc^R^	This study
*Sphingomonas aquatilis*	Wild type, non-nicotine-degrading strain, G^−^, Tc^S^	Lee et al., 2001
A0463 pRK415*ndrA1A2A3*	*Sphingomonas aquatilis* transformed with pRK415-*ndrA1A2A3*, Tc^R^	This study
A0463 pRK415-*ndrB1B2B3B4*	*Sphingomonas aquatilis* transformed with pRK415*-ndrB1B2B3B4*, Tc^R^	This study
A0463 pRK415-*ndrB1B2B3B4orf1orf2*	*Sphingomonas aquatilis* transformed with pRK415-*ndrB1B2B3B4orf1orf2*, Tc^R^	This study
A0463pRK415	*Sphingomonas aquatilis* transformed with pRK415, Tc^R^	This study
*Pseudomonas putida* species		
KT2440	Metabolically versatile saprophytic soil bacterium	Nelson et al., 2002
KT2440 pRK415-*ndrA1A2A3*	KT2440 transformed with pRK415-*ndrA1A2A3*, Tc^R^	This study
KT2440pRK415-*ndrB1B2B3B4*	KT2440 transformed with pRK415- *ndrB1B2B3B4*, Tc^R^	This study
KT2440pRK415 *ndrB1B2B3B4orf1orf2*	KT2440 transformed with pRK415-*ndrB1B2B3B4orf1orf2*, Tc^R^	This study
KT2440pRK415	KT2440 transformed with pRK415, Tc^*R*^	This study
**PLASMIDS**		
pTnMod-Okm	Source of kanamycin resistance gene	Dennis and Zylstra, 1998
pEX18Tc	Gene knockout vector, *oriT*^+^, *sacB*^+^, Tc^*R*^	Hoang et al., 1998
pEX18Tc-*ndrA1*	*ndrA1* gene knockout vector containing two DNA fragments homologous to the upstream and downstream regions of the *ndrA1* and kanamycin resistance gene from pTnMod-Okm	This study
pEX18Tc-*ndrB2*	*ndrB2* gene knockout vector containing two DNA fragments homologous to the upstream and downstream regions of the *ndrB2* and kanamycin resistance gene from pTnMod-Okm	This study
pRK415	Broad host range vector, Tc^R^	Keen et al., 1988
pRK415-*ndrA1*	*ndrA1* gene complementation vector by fusing *ndrA1* into the *Hin*d III-*Eco*R I restriction site of pRK415	This study
pRK415-*ndrB2*	*ndrB2* gene complementation vector by fusing *ndrB2* into the *Hin*d III-*Eco*R I restriction site of pRK415	This study
pRK415-*ndrA1A2A3*	Heterologous expression vector with *ndrA1A2A3* insert into the *Hin*d III-*Eco*R I restriction site of pRK415	This study
pRK415-*ndrB1B2B3B4*	Heterologous expression vector with *ndrB1B2B3B4* insert into the *Hin*d III-*Eco*R I restriction site of pRK415	This study
pRK415-*ndrB1B2B3B4orf1orf2*	Heterologous expression vector with *ndrB1B2B3B4orf1orf2* insert into the *Hin*d III-*Eco*R I restriction site of pRK415	This study
pET-28a(+)	Expression vector, Kan^R^, C/N-terminal His·Tag/thrombin/T7·Tag, T7 *lac* promoter, T7 transcription start, f1 origin, *lacI*	Novagen
pET28a-*ndrB1B2B3B4orf1orf2*	Expression vector for *ndrB1B2B3B4orf1orf2* with C-terminal His·Tag by cloning *ndrB1B2B3B4orf1orf2* into the *Nco* I-*Hin*d III restriction site	This study

### Draft genome sequencing of *S. melonis* TY and prediction of relevant nicotine metabolism genes

The draft genome sequence of *S. melonis* TY was obtained using Illumina Hiseq2000 paired-end (PE) sequencing (101 bp for each read; 299.8-fold coverage) and then assembled into 68 scaffolds using SOAPdenovo version 2.04 (N50 length, 205,244 bp) (Li et al., 2010). The draft genome sequence of strain TY contains 4,100,783 bp with a GC content of 67.083% and 3748 predicted coding sequences (CDSs). The annotation was performed using Best-placed reference protein set; GeneMarkS+. Previously described nicotine-degrading genes, including *ndhLSM*, which catalyzes the first step of nicotine transformation in *A. nicotinovorans* (Dang Dai et al., 1968; Grether-Beck et al., 1994), and *vppA* and *vppB* of the upper VPP pathway of *Ochrobactrum* sp. SJY1 (Yu et al., 2014, 2015), were used for blast searches of the strain TY genome in NCBI.

### RT-qPCR analysis of *ndrA1A2A3* and *ndrB1B2B3B4*

RNAprep Pure Bacteria Kit (Tiangen Biotech, Beijing, China) was used to prepare total RNA from *S. melonis* TY grown in triplicate in control and nicotine-induction cultures. The RNA was reverse transcribed into cDNA using random hexamer primers and PrimeScript RT Reagent Kit with gDNA Eraser (Perfect Real Time) (Takara, Dalian, China). The respective cDNA fragments were used as templates in PCR with gene-specific primers (Table S1). Real-time quantitative PCR was performed using a Rotor-Gene Q real-time PCR detection system (Qiagen) with TransStart Top Green qPCR SuperMix (TransGen Biotech, Beijing, China). The strain TY genome was used as the positive control, and untranscribed RNA was used as the negative control. Melting curves and agarose gel analyses were applied to confirm the specificity of the PCR products. The threshold cycle (C_T_) values for each target gene were normalized to the V3 region of the 16S rRNA gene. The 2^−ΔΔCT^ method was used to calculate the relative expression level, where ΔΔC_T_ = (C_T, target_-C_T, 16S_)_induction_—(C_T, target_-C_T, 16S_)_control_; to obtain more reliable results, the theoretical efficiency value 2 was replaced with the estimated PCR efficiency value (Livak and Schmittgen, 2001; Ramakers et al., 2003; Ruijter et al., 2009; Tuomi et al., 2010).

### Gene knockout and complementation of *ndrA1* and *ndrB2*

In-frame disruption of *ndrA1* and *ndrB2* in strain *S. melonis* TY was performed using the suicide plasmid pEX18Tc and a two-step homologous recombination method as described previously (Chen et al., 2014). Gene complementation were conducted the same as described in Chen et al. (2014).

### TYΔ*ndrA1* and TYΔ*ndrB2* growth and resting cell reactions

Biotransformation tests were performed using wild-type TY and derivative strains TYΔ*ndrA1*, TYΔ*ndrA1*(pRK415- *ndrA1*), TYΔ*ndrB2*, and TYΔ*ndrB2*(pRK415-*ndrB2*) as well as an inactivated wild-type TY strain [heated at 80°C for 5 min to prevent nicotine absorption (Harwood et al., 1994)] and a control consisting only of nicotine. The biotransformation test was performed in a 150 mL flask containing 60 mg dry cell weight of resting cells (approximately 5 OD_600nm_, with one OD_600nm_ unit equal to 0.40 g/L dry cell weight) and 0.5 g/L nicotine in 30 mL of sterilized ISM medium; the culture was incubated at 30°C and 200 rpm. Three sets of parallel experiments were performed for each strain. Biotransformation tests using 6 HN were conducted in a manner similar to nicotine that described above.

To test whether the two mutant strains retained the ability to metabolize 6 HN, the first product formed in the VPP pathway, strains TYΔ*ndrA1*, and TYΔ*ndrB2* were washed three times with sterilized water and streaked onto ISM plates supplemented with 6 HN as the sole source carbon and nitrogen; the plates were placed in an incubator (30°C) for growth; wild-type TY was used as a positive control.

### Heterologous expression of two sets of putative nicotine-degrading genes

Nicotine catalysis activity of *ndrA1A2A3* and *ndrB1B2B3B4* was tested by heterologous expression. To test for nicotine dehydrogenase activity using whole cell or crude cell extracts, the two gene clusters were cloned (using primers shown in Table S1) into the broad host-range cloning vector pRK415 (*Hin*d III- and *Eco*R I digested) and mated to *P. putida* KT2440 and *Sphingomonas aquatilis* (two non-nicotine-degrading strains) via *E. coli* WM3064. The culture conditions and enzyme activity detection are those previously reported (Ganas and Brandsch, 2009), while the detection of production 6 HN was replaced by UV-scan, and the host transformed with the control plasmid was used as the negative control, wild-type TY set as positive control.

Genes *orf2* (coding for glycosyl transferase) and *orf1* (coding for a XshC-Cox1-family protein) just downstream of *ndrB4* exhibit similarity with *mobA* and *coxF*, respectively, which are essential for nicotine dehydrogenase activity in *A. nicotinovorans* pAO1 (Ganas and Brandsch, 2009) and may be required for nicotine dehydrogenase cofactor synthesis in strain TY. We cloned *orf1* and *orf2* together with *ndrB1B2B3B4* into vector pRK415 (*Hin*d III- and *Eco*R I digested) according to a previous report (Ganas and Brandsch, 2009), followed by transformation of the plasmid into *E. coli* DH5α and mating to *S. aquatilis* and *P. putida* KT2440 via *E. coli* WM3064. Moreover, we also cloned these six genes into vector pET-28a(+) (*Hin*d III- and *Nco* I-digested) just downstream the T7 promoter and transformed the plasmid into *E. coli* BL21(DE3) to induce expression with various concentrations of IPTG (at 0, 0.1, 0.2, 0.5 and 1 mM). Activity tests were performed using whole cell and crude cell extracts of the above recombinant strains and corresponding negative controls with the host containing the original plasmid, pRK415 or pET-28a(+). SDS-PAGE was carried out for the *E. coli* BL21(DE3) derivative strains to detect induction under the test conditions.

### Genome sequence accession number

This whole-genome shotgun project has been deposited at DDBJ/ENA/GenBank under accession LQCK00000000. The version described in this paper is version LQCK02000000.

## Results

### Two sets of putative nicotine dehydrogenase genes in strain TY

**Figure 1:**
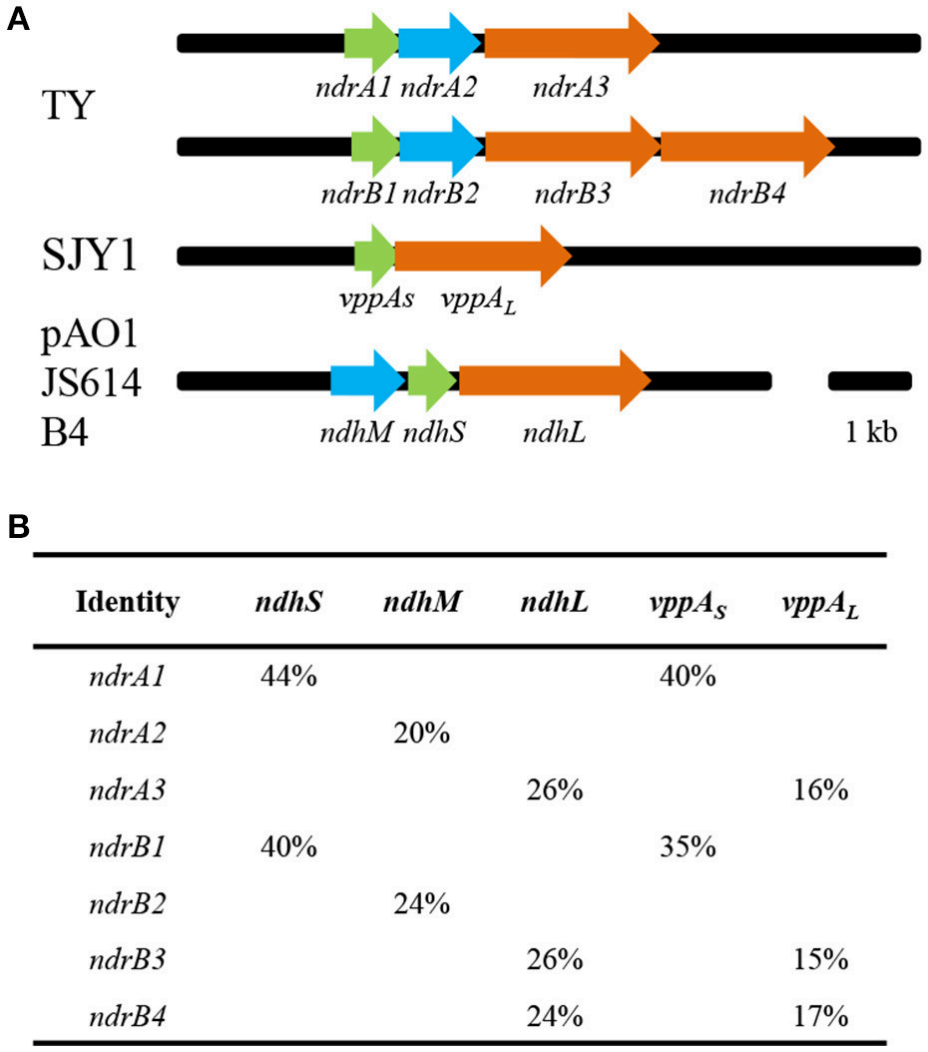
**Genetic organization of clusters ***ndrA1A2A3*** and ***ndrB1B2B3B4*** in ***Sphingomonas melonis*** TY and amino acid sequence identity with related genes (A)**. Arrows indicate the size and direction of transcription of each gene. *vppAs*, nicotine hydroxylase, subunit S; *vppA*_*L*_, nicotine hydroxylase, subunit L; *ndhM*, nicotine dehydrogenase, subunit M; *ndhS*, nicotine dehydrogenase, subunit S; *ndhL*, nicotine dehydrogenase, subunit L. TY, *Sphingomonas melonis* TY; SJY1, *Ochrobactrum* sp. SJY1; pAO1, *A. nicotinovorans* pAO1; JS614, *Nocardioides* sp. JS614; B4, *Rhodococcus opacus* B4. **(B)** Comparison of amino acid sequences; the percentage shows the amino acid identity.

Genome sequencing can provide much information for research into the functional mechanisms of microorganisms (Tang et al., 2013). The draft genome sequence of *S. melonis* TY was obtained by Illumina sequencing, and blast searching of the sequence using representative sequences for nicotine-degrading enzymes from *A. nicotinovorans* and *Ochrobactrum* sp. SJY1 was performed. Based on gene similarity and organization, we identified two sets of putative nicotine dehydrogenase genes in TY: *ndrA1A2A3* and *ndrB1B2B3B4*. According to the annotation of genome sequence, *ndrA1* encodes an aldehyde dehydrogenase iron-sulfur subunit; *ndrA2* encodes a subunit of molybdopterin dehydrogenase; *ndrA3* encodes a subunit of xanthine dehydrogenase; *ndrB1* encodes an aldehyde dehydrogenase iron-sulfur subunit; *ndrB2, ndrB3*, and *ndrB4* all encode subunit of xanthine dehydrogenase. These two sets of gene clusters, *ndrA1A2A3* and *ndrB1B2B3B4*, show similarity to the three subunits of *ndh*: nicotine dehydrogenase subunit S (*ndhS*), nicotine dehydrogenase subunit M (*ndhM*) and nicotine dehydrogenase subunit L (*ndhL*). *ndrA3, ndrB3*, and *ndrB4* show similarity to nicotine hydroxylase subunit L (*vppA*_*L*_), and *ndrA1* and *ndrB1* show similarity to nicotine hydroxylase subunit S (*vppA*_*S*_). All these genes exhibit 15–44% amino acid sequence identity with homologous nicotine-degrading proteins, with similar gene organization. The gene organization and amino acid identity shared with homologous genes *ndh* and *vppA* are shown in Figure 1.

### Transcriptional levels of *ndrA1A2A3* and *ndrB1B2B3B4* are upregulated in the presence of nicotine

**Figure 2:**
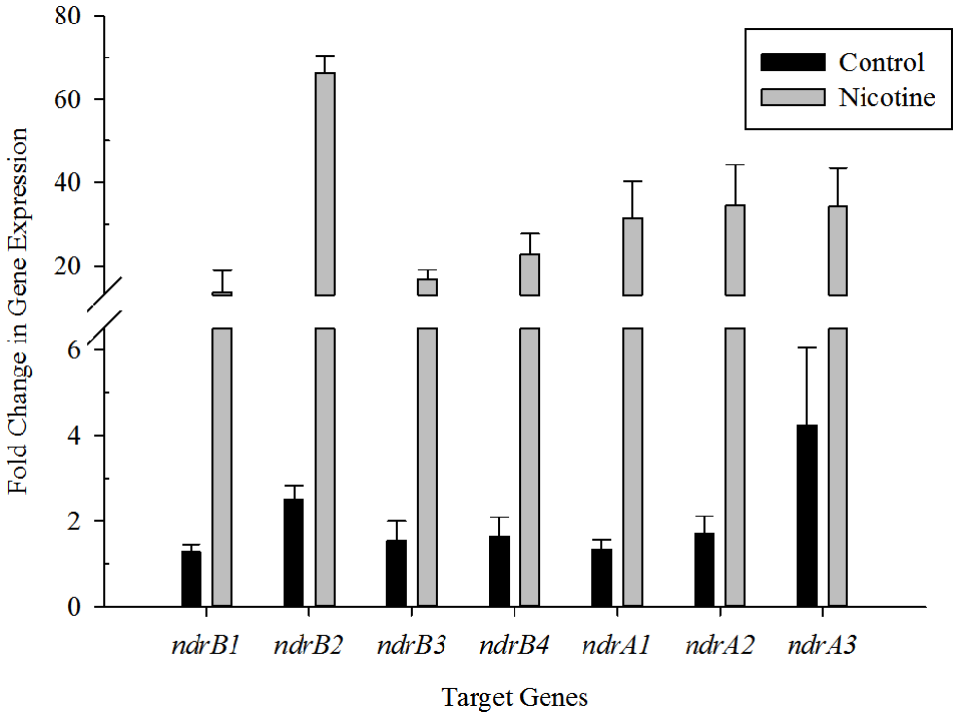
**Changes in transcriptional levels of genes putatively involved in nicotine degradation**. RT-qPCR analysis of target gene transcripts produced in *Sphingomonas melonis* TY grown with or without nicotine. The results presented in these histograms are the means of three independent experiments, and error bars indicate the standard error.

To demonstrate a correlation between nicotine degradation and the two sets of putative nicotine dehydrogenase genes, the levels of mRNA expression of seven putative target genes involved in nicotine degradation in *S. melonis* TY were estimated using RT-qPCR and the 2^ΔΔCT^ method in the presence or absence of nicotine. The V3 region of the 16S rRNA gene was used as the reference. The results showed that transcription of these genes was significantly upregulated in the presence of nicotine compared to in its absence (Figure 2), suggesting that transcription of *ndrA1A2A3* and *ndrB1B2B3B4* was induced by nicotine.

### The identification and characterization of *ndrA1A2A3* and *ndrB1B2B3B4*

**Figure 3:**
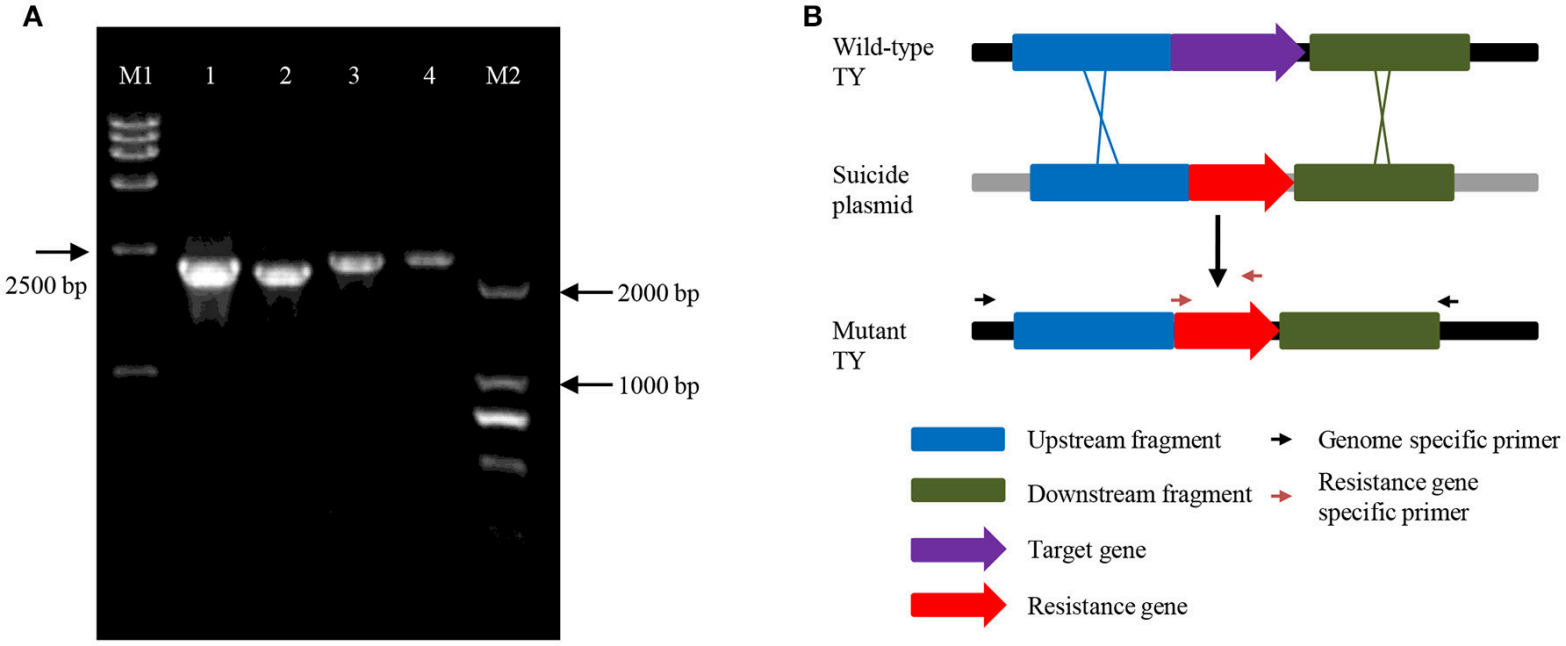
**PCR verification of TYΔ***ndrA1*** and TYΔ***ndrB2*** using specific primers beyond the homologous fragments and primers in the resistance gene. (A)** Sample 1, amplified with ndrA1-VF and Kan02, 2260 bp; sample 2, amplified with ndrA1-VR and Kan01, 2172 bp; sample 3, amplified with ndrB2-VF and Kan01, 2308 bp; sample 4, amplified with ndrB2-VR and Kan02, 2330 bp; M1, DL15000 marker; M2, DL2000 marker. Electrophoresis was performed using 0.8% agarose; **(B)** The in-frame deletion of target genes, and relative site of genomic-specific primers and resistance gene-specific primers.

**Figure 4:**
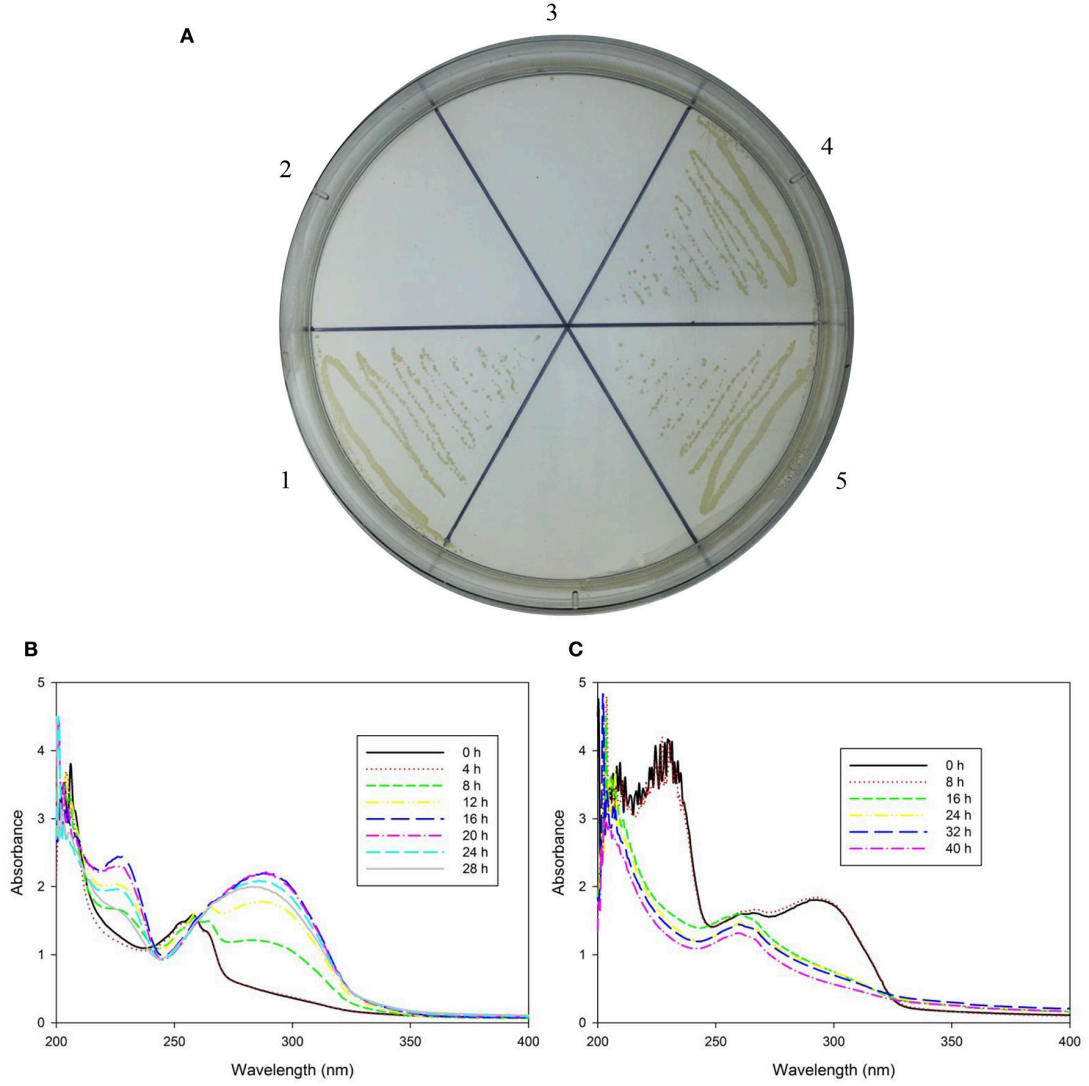
**Growth ability of strain TY and its derivatives with nicotine and their biotransformation ability with nicotine and 6-hydroxynicotine**. **(A)** Growth ability of wild-type TY (1), TYΔ*ndrA1* (2), TYΔ*ndrB2* (3), TYΔ*ndrA1*(pRK415-*ndrA1*) (4), and TYΔ*ndrB2*(pRK415-*ndrB2*) (5); **(B)** Biotransformation of nicotine by wild-type TY and two complementary strains, and example is shown because they are similar spectrometric graphs; **(C)** Biotransformation of 6-hydroxynicotine by wild-type TY, two mutant strains and two complementary strains, example is shown because they are similar spectrometric graphs.

To examine whether *ndrA1A2A3* or *ndrB1B2B3B4* is involved in nicotine degradation *in vivo, ndrA1* or *ndrB2* was knocked out via kanamycin resistance gene insertion using a gene replacement technique based on homologous recombination. Double crossover was verified by PCR (Figure 3) and sequencing analysis. Although neither mutant strain, TYΔ*ndrA1*, or TYΔ*ndrB2*, could grow on ISM containing nicotine as the sole carbon and nitrogen source (Figure 4), both mutants were able to grow on ISM supplemented with 1 g/L (NH_4_)_2_SO_4_ and 1 g/L glucose as the nitrogen and carbon sources, respectively. Furthermore, following complementation of the corresponding gene, each mutant displayed restored capacity to grow on nicotine, similar to wild-type TY (Figure 4).

Based on the results of gene knockout and complementation, it appears that *ndrA1* and *ndrB2* are involved in the capability of strain TY to utilize nicotine as a sole carbon and nitrogen source, suggesting that one of these two sets of gene clusters encodes a nicotine dehydrogenase. To further confirm the function of *ndrA1A2A3* and *ndrB1B2B3B4*, we performed a nicotine and 6 HN biotransformation test using the wild-type TY strain, the mutant strains and strains complemented with the two genes. Although TYΔ*ndrA1* and TYΔ*ndrB2* lost the ability to degrade and transform nicotine, they retained 6 HN conversion capacity (Figure 4). Complementation of the corresponding gene restored the ability of both TYΔ*ndrA1*(pRK415-*ndrA1*) and TYΔ*ndrB2*(pRK415-*ndrB2*) to degrade and transform nicotine.

**Figure 5:**
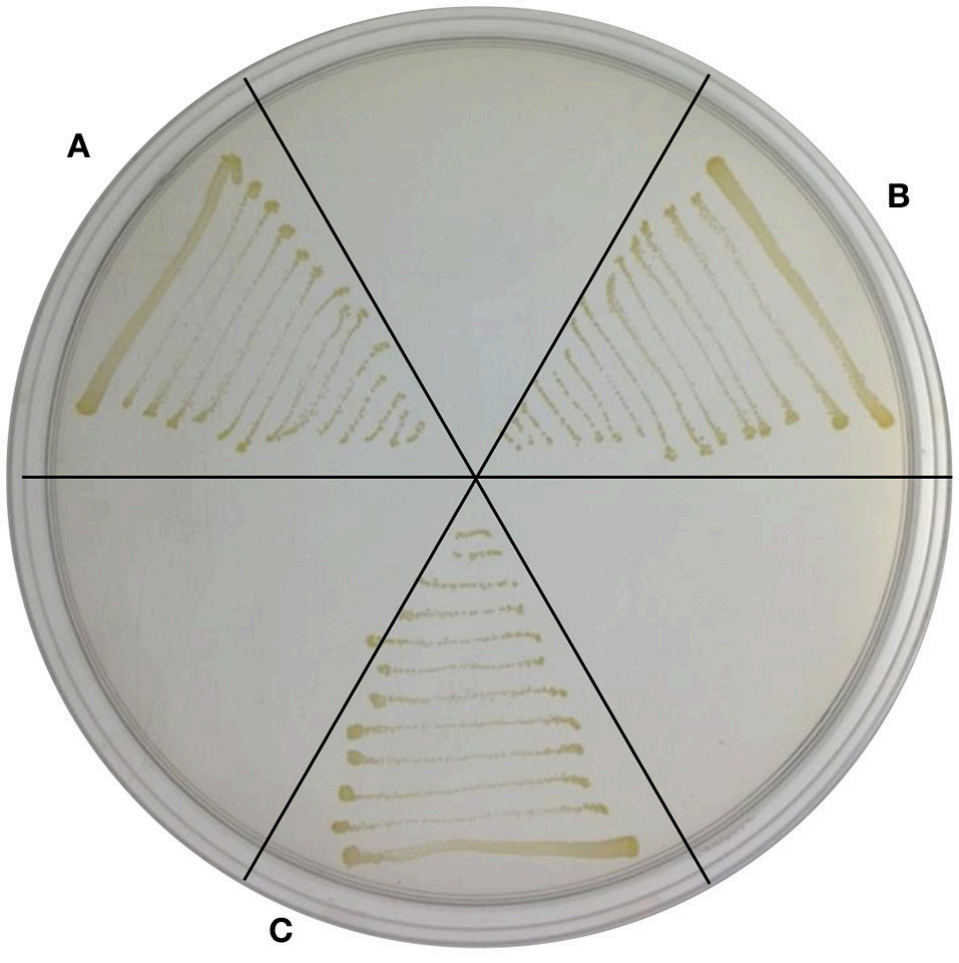
**Growth of TYΔ***ndrA1*** and TYΔ***ndrB2*** in ISM supplemented with 6-hydroxynicotine as a substrate**. **(A)**, TYΔ*ndrA1*; **(B)**, TYΔ*ndrB2*; **(C)**, wild-type TY.

Because TYΔ*ndrA1* and TYΔ*ndrB2* can grow on ISM medium supplemented with 6 HN as the sole carbon and nitrogen source (Figure 5), combined with the biotransformation results, it appears that the two mutant strains not only retain the ability to transform 6 HN but can also degrade it completely. This result indicates that disruption of *ndrA1* or *ndrB2* only affects the first step in nicotine metabolism, supporting our speculation that a nicotine dehydrogenase is encoded by one of these two sets of genes.

### No catalytic activity was detected after heterologous expression of *ndrA1A2A3* and *ndrB1B2B3B4*

To shed light on the function of these two gene clusters, we tested the nicotine-degrading activity of *ndrA1A2A3* and *ndrB1B2B3B4* through heterologous expression in *P. putida* KT2440 and *S. aquatilis*; the latter was selected because it is a member of the same genus as strain TY but does not exhibit nicotine degradation ability. However, neither whole cell nor crude cell extracts exhibited nicotine dehydrogenase activity.

The cofactor molybdopterin cytosine dinucleotide (MCD) is needed for Ndh nicotine dehydrogenase function in *A. nicotinovorans* pAO1 (Sachelaru et al., 2006; Ganas and Brandsch, 2009), we thought MCD or other cofactor is needed by the putative nicotine dehydrogenase in strain TY. Thus, we identified two genes, *orf1* and *orf2*, contiguous on the genome with *ndrB1B2B3B4* that show similarity with *coxF* and *mobA* (involved in MCD biosynthesis in *A. nicotinovorans* pAO1), and *orf2* shows LAAG amino acid motif as the canonical MocA (Neumann et al., 2011). We cloned these six genes into pRK415 for expression in *E. coli* DH5α, *S. aquatilis* and *P. putida* KT2440 and into pET-28a(+) for expression in *E. coli* BL21(DE3). However, whole cell and crude cell extracts showed no nicotine dehydrogenase activity. In addition, SDS-PAGE was carried out on extracts from *E. coli* BL21(DE3)pET28a-*ndrB1B2B3B4orf1orf2* and its control strain *E. coli* BL21(DE3)pET28a, and several bands were appeared in the former compared to the latter (data not shown).

## Discussion

*S. melonis* TY can degrade nicotine efficiently than many other nicotine degraders, and has tolerance to some neonicotinoid insecticides when degrading nicotine, which is suitable for the disposal of tobacco waste and the reduction of nicotine in tobacco leaves and this tolerance was not reported in other nicotine degraders (Wang et al., 2011). In this study, we identified two sets of genes involved in the nicotine metabolism in *S. melonis* TY. Both mutants were able to grow on glucose and (NH_4_)_2_SO_4_, indicating that disruption of *ndrA1* and *ndrB2* did not affect basic metabolism. Moreover, following gene complementation, both complemented strains regained the ability to utilize nicotine. The gene knockout and complementation results indicated that *ndrA1* and *ndrB2* may be involved in converting nicotine to 6 HN, the first step in the VPP pathway.

To elucidate the function of these two gene clusters, we tested the nicotine-degrading activity of *ndrA1A2A3* and *ndrB1B2B3B4*. Many gene products show activity when heterologously expressed in *E. coli* DH5α and *P. putida* KT2440, such as the nicotine hydroxylation *vppA* gene from *Ochrobactrum* sp. strain SJY1 (Yu et al., 2015), the 6 HN oxidation *6 hlno* gene from *A. nicotinovorans* pAO1 (Ganas and Brandsch, 2009), and the nicotine oxidoreduction *nicA* gene from *P. putida* S16. Furthermore, homologous expression is an effective approach for successful protein expression, for example, *6 hlno* and *ndhMSLcoxFmobA* (for nicotine oxidoreduction) (Ganas and Brandsch, 2009) and *ndhL* and *kdhL* (for 6 HPON dehydrogenation) (Sachelaru et al., 2006). However, expression of *ndrA1A2A3* and *ndrB1B2B3B4* in *P. putida* KT2440 and *S. aquatilis* did not confer activity on a heterologous host. Based on nicotine-related research of well-established *A. nicotinovorans*, we speculate that the nicotine dehydrogenase in strain TY might require a cofactor, such as MCD in *A. nicotinovorans* (Sachelaru et al., 2006). Surprisingly, we found two genes showing similarity to *mobA* and *coxF* contiguous with *ndrB1B2B3B4* on the strain TY genome. We cloned these two genes, *orf1* and *orf2*, together with *ndrB1B2B3B4* into vectors pRK415 and pET-28a(+) and transformed the plasmids into *E. coli* DH5α, *S. aquatilis, P. putida* KT2440, and *E. coli* BL21(DE3). However, no nicotine dehydrogenase activity was detected. Indeed, heterologous expression with a constitutive or inducible promoter showed that *ndrA1A2A3* and *ndrB1B2B3B4* alone cannot confer nicotine dehydrogenase activity. Considering that TYΔ*ndrA1* and TYΔ*ndrB2* retained the ability to degrade 6 HN and that *ndrA1A2A3* and *ndrB1B2B3B4* alone could not confer nicotine dehydrogenase activity, we propose that *ndrA1A2A3*, and *ndrB1B2B3B4* are involved and necessary for nicotine dehydrogenase in strain TY but require additional factors. Additionally, disruption of *ndrA1* or *ndrB2* resulted in no nicotine dehydrogenase activity.

There are possible two speculations for the reasons why the expression of the *ndrA1A2A3* and *ndrB1B2B3B4* in the tested strains failed. First, the tested strains did not provide a suitable environmental for the transcription, translation or protein folding of the introduced gene sets, such as the different codon usage bias between the gene sets in strain TY and the tested strains and any incorrect step in the formation of functional protein. These errors will lead to the negative results. Second, these two gene sets may take no part in catalyzing the initial degradation of nicotine but perform other essential function in nicotine degradation in strain TY. However, we think that influence of uptake of nicotine by the tested strains can be excluded because both whole cell and crude cell extracts reactions were performed to exhibit activities, and *P. putida* KT2440 were reported to express functional *vppA* (Yu et al., 2015).

In studying related genes involved in certain pathway or cellular network, there are several approaches for generating mutants. Nonetheless, some mutants obtained are in genes that do not actually function in the studied pathway due to the influence of gene organization, such as polar effects on downstream genes. However, *ndrA1A2A3* and *ndrB1B2B3B4* are not contiguous in the strain TY genome, and there are no other nearby putative genes related to nicotine degradation. Knockout of *ndrA1* and *ndrB2* led to the same superficial phenotypic effect on the undefined nicotine dehydrogenase in strain TY, which was presumably not due to potential downstream effects. Generally, we thought the function of both clusters *ndrA1A2A3* and *ndrB1B2B3B4* is essential for nicotine degradation in strain TY.

In a word, current experimental data only support that *ndrA1A2A3* and *ndrB1B2B3B4* are essential in nicotine degradation in strain TY, and the relationship between them is unclear. We have proposed three hypotheses about the relationship between these two gene sets. First, because the details of the reaction mechanism of nicotine conversion to 6 HN are not available and it is classified as different type of reaction according to the source of oxygen in hydroxylation (Hirschberg and Ensign, 1971), and the enzyme is considered as hydroxylase or oxygenase accordingly.. Moreover, reports have speculated some similar mechanisms in nicotinic acid and nicotine degradation (Tang et al., 2012). A previous study showed that 6-hydroxynicotinic acid is formed during nicotinic acid degradation; moreover, the oxygen incorporated into 6-hydroxynicotine acid was from water and not oxygen gas (Hunt et al., 1958; Hirschberg and Ensign, 1971). It was proposed that a dihydro-monohydroxy derivative of nicotinic acid was formed during nicotinic acid degradation, though no such derivative was identified (Hunt et al., 1958). Combining the similarity of the pyridine ring structure and the similar hydroxylation reaction of the pyridine ring in nicotinic acid and nicotine, we speculate that the same molecular mechanism of hydroxylation occurs with nicotine to generate 6 HN, as it does with nicotinic acid. If a type of dihydro-monohydroxy derivative of nicotine is generated, then *ndrA1A2A3* and *ndrB1B2B3B4* may play essential roles in converting nicotine to its dihydro-monohydroxy derivative and then to 6 HN. Second, one of the two sets of gene is directly related with nicotine degradation and need specific cofactor, and the other one is indirectly related. Third, neither of the two sets of genes is direct related with nicotine degradation, and there is an undefined real nicotine dehydrogenase in strain TY. However, this kind of undirected related function is unknown and need further study.

In summary, this study provides evidence indicating that two novel sets of genes in *S. melonis* TY are crucial for nicotine degradation. These two sets of genes are first reported here as being involved in the variant of the pyridine and pyrrolidine pathway of nicotine degradation and show similarity with isoenzymes in other nicotine-degrading microbes, an unexpected and surprising result. It will be important to determine whether *ndrA1A2A3* and *ndrB1B2B3B4* are both involved and/or function together in nicotine dehydrogenase activity as well as what type of reactions are involved in the still-undefined nicotine dehydrogenase in strain TY. Although uncertainty remains regarding the exact function of these two gene clusters, further research is in progress to characterize their function(s). These findings will provide a deeper understanding of the molecular mechanisms of nicotine metabolism in *Sphingomonas*.

## Ethics statement

This article does not contain any studies with human participants or animals performed by any of the authors.

## Author contributions

Revising the work: CX, NZ and ZL. Wrote the paper and final approval of the version to be published: All authors. All authors agreed to be accountable for all aspects of the work in ensuring that questions related to the accuracy or integrity of any part of the work are appropriately investigated and resolved.

## Funding

This work was financially supported by the National Natural Science Foundation of China (No.31170115 and 31422003).

### Conflict of interest statement

The authors declare that the research was conducted in the absence of any commercial or financial relationships that could be construed as a potential conflict of interest.
