# Supplementary material for: Two Novel Sets of Genes Essential for Nicotine Degradation by Sphingomonas melonis TY
Source: Front Microbiol. 2017 Jan 17;7:2060. doi: 10.3389/fmicb.2016.02060 (PMC5239795; doi:10.3389/fmicb.2016.02060)
Supplement: Supplementary file 1 [file Table1.DOCX]

**Article Title:**

**Two novel sets of dehydrogenase genes essential for nicotine degradation by *Sphingomonas* *melonis* TY**

**Journal name:**

Frontiers in Microbiology

**Author names:**

Haixia Wang^1^, Panpan Zhu^1^, Ning-Yi Zhou^2^ and Zhenmei Lu^1*^

**Affiliation and E-mail:**

^1^Institute of Microbiology, College of Life Sciences, Zhejiang University, Hangzhou, China

^2^State Key Laboratory of Microbial Metabolism & School of Life Sciences and Biotechnology, Shanghai Jiao Tong University, Shanghai, China

* Zhenmei Lu

[lzhenmei@zju.edu.cn](mailto:lzhenmei@zju.edu.cn)

**TableS1** Oligonucleotide primers used in this study

| Primer name | Primer sequence (5’-3’) | Purpose |
| --- | --- | --- |
| Gene knockout and complementation | | |
| Kan01 | tgtctcaaaatctctgatgttac | To amplify the kanamycin resistance gene from pTnMod-Okm for gene knockout |
| Kan02 | ttagaaaaactcatcgagcatc |  |
| ndrA1 upF^a^ | ggccagtgccaagctaccaccgactacgacaacaacagc | To amplify an upstream fragment of *ndrA1* for gene knockout |
| ndrA1 upR | agagattttgagacacaaaggccccttgatgcatttg |  |
| ndrA1downF | gatgagtttttctaaaggccttcacctacgaacgcg | To amplify a downstream fragment of *ndrA1* for gene knockout |
| ndrA1downR | atgattacgaattcggctgccttcaccgctgctgtc |  |
| ndrB2 upF | atgattacgaattcgagcgggacgaataggggatgtt | To amplify an upstream fragment of *ndrB2* for gene knockout |
| ndrB2 upR | gatgagtttttctaaggcatgttcggtttcggtaagg |  |
| ndrB2downF | agagattttgagacagcctctgcctccgccttggtgt | To amplify a downstream fragment of *ndrB2* for gene knockout |
| ndrB2downR | ggccagtgccaagctgttccttggaccgccgcttgg |  |
| pEX18Tc-VF | gcacgacaggtttcccgactg | To verify construction of pEX18Tc-related constructs by PCR or sequencing |
| pEX18Tc-VR | ccgcttctgcgttctgattta |  |
| ndrA1-VF | ccgcagtcgctgtggtggaa | To verify strain TYΔ*ndrA1* by PCR or sequencing |
| ndrA1-VR | tctggtcgggcgtctcgtag |  |
| ndrB2-VF | ttggcggcggacttcat | To verify strain TYΔ*ndrB2* by PCR or sequencing |
| ndrB2-VR | cataggtcccgatttgcttca |  |
| ndrA1-CF | tgattacgccaagcttcatgagcgattacgaactgtcca | To amplify *ndrA1* for gene complementation |
| ndrA1-CR | gacggccagtgaattctcatgctttggctcccgcgaca |  |
| ndrB2-CF | tgattacgccaagcttcatgaagaccttcacctatgcca | To amplify *ndrB2* for gene complementation |
| ndrB2-CR | gacggccagtgaattcttattctcccgtcaggtcgcga |  |
| pRK415-VF | gcccaatacgcaaaccgcct | To verify construction of pRK415-related complementation plasmids by PCR or sequencing |
| pRK415-VR | gctctcctgttccgaccctg |  |
| RT-qPCR | | |
| RT-ndrA1F | gacgagatcatcacgatc | To amplify a 98 bp fragment of *ndrA1* for qPCR |
| RT-ndrA1R | caataaccgcactgatag |  |
| RT-ndrA2F | atacgccgcatatcgaaa | To amplify a 102 bp fragment of *ndrA2* for qPCR |
| RT-ndrA2R | cacgcaccttatgatagatg |  |
| RT-ndrA3F | cttcttcgtcaaccatga | To amplify a 180 bp fragment of *ndrA3* for qPCR |
| RT-ndrA3R | gcattgtagatcgcattg |  |
| RT-ndrB4F | gacttcggcgaatatcac | To amplify a 162 bp fragment of *ndrB4* for qPCR |
| RT-ndrB4R | gttgtagatggcgttgat |  |
| RT-ndrB3F | atgttcggtttcggtaag | To amplify a 196 bp fragment of *ndrB3* for qPCR |
| RT-ndrB3R | ggactagatagccgtagg |  |
| RT-ndrB2F | gtatctccatccacgaatt | To amplify a 163 bp fragment of *ndrB2* for qPCR |
| RT-ndrB2R | atcgcaaaggcataagac |  |
| RT-ndrB1F | ctatcagtgcggctattg | To amplify a 187 bp fragment of *ndrB1* for qPCR |
| RT-ndrB1R | atcgacgatgttgggata |  |
| 338F | cctacgggaggcagcagcag | To amplify the V3 region of the 16S RNA gene |
| 518R | attaccgcggctgctgg |  |
| Heterologous expression |  |  |
| pRK415-ndrA1A2A3F | tgattacgccaagcttcatgagcgattacgaactgtcc | To amplify *ndrA1A2A3* for gene expression |
| pRK415- ndrA1A2A3R | gacggccagtgaattcttacgccaccttcgccatgcc |  |
| pRK415-ndrB1B2B3B4F | tgattacgccaagcttcatgcacttcacgatcaacgg | To amplify *ndrB1B2B3B4* for gene expression |
| pRK415- ndrB1B2B3B4R | gacggccagtgaattcttagagagcgggcaagcccg |  |
| pRK415-ndrB1B2B3B4orf1orf2F | tgattacgccaagcttcatgcacttcacgatcaacgg | To amplify an upstream region of *ndrB1B2B3B4orf1orf2* for gene expression |
| pRK415- ndrB1B2B3B4orf1orf2MR | gagggcgaatgtaaggtgttgg |  |
| pRK415- ndrB1B2B3B4orf1orf2 MF | gcccaacaccttacattcgc | To amplify a downstream region of *ndrB1B2B3B4orf1orf2* for gene expression |
| pRK415-ndrB1B2B3B4orf1orf2R | gacggccagtgaattcttacgaccgccgaaggcggg |  |
| pET28a-ndrB1B2B3B4orf1orf2F | agaaggagatataccatgcacttcacgatcaacgg | Paired with pRK415-ndrB1B2B3B4orf1orf2MR to amplify an upstream region of *ndrB1B2B3B4orf1orf2* for gene expression |
| pET28a-ndrB1B2B3B4orf1orf2R | gtgcggccgcaagcttttacgaccgccgaaggcgggt | Paired with pRK415-ndrB1B2B3B4orf1orf2 MF to amplify a downstream region of *ndrB1B2B3B4orf1orf2* for gene expression |

^a^: A 15 bp overlap (5’) in the sequences of the primers for in-fusion cloning is underlined.
